# Promoting school lunch fruit and vegetable intake through role modeling: a pilot study

**DOI:** 10.3934/publichealth.2020002

**Published:** 2020-01-07

**Authors:** Stephanie S Machado, Michael Burton, Wes Loy, Kyle A Chapman

**Affiliations:** 1 School of Public Health, University of California, Berkeley, Berkeley, CA, United States; 2 Department of Humanities and Social Sciences, Oregon Institute of Technology, Klamath Falls, OR, United States

**Keywords:** United States, role modeling, fruits and vegetables, schools, intervention

## Abstract

**Objectives:**

Child fruit and vegetable consumption is a critical component of adult chronic disease prevention, yet fruit and vegetable intake remains low among elementary school children in the United States. This pilot study tested a role modeling intervention designed to promote fruit and vegetable consumption in a U.S elementary school cafeteria setting.

**Methods:**

This one-year, repeated cross-sectional study used digital photographs to assess fruit and vegetable waste at baseline (n = 566 trays) and follow-up (n = 231 trays) of kindergarten through fifth grade students in one elementary school. Differences in waste were assessed through Mann-Whitney statistical tests. Feedback on intervention acceptability was provided by the intervention team during implementation.

**Results:**

The proportion of students consuming all of their selected fruits and vegetables increased by 11.1% and 8.7% respectively (p < 0.01). There was a significant decrease in the proportion of students not consuming any of their selected fruit (16.0%, p < 0.001). Staff and students provided positive reports of intervention acceptance.

**Conclusions and Implications:**

Findings from this pilot study indicate that role modeling in a school cafeteria setting may be a promising health promotion strategy and provide groundwork for future research in the development of school cafeteria role modeling interventions. Further research is needed to assess intervention efficacy and acceptability at a larger scale.

## 1. Introduction

Fruits and vegetables are critical to health across the lifespan. Intake is especially important during childhood, as children who consume little fruits or vegetables are at greater risk of developing diabetes [1] and cardiovascular disease [2] in adulthood. Despite this evidence, American elementary school-aged children do not meet the United States Healthy People 2020 target for fruits and vegetables [3].

In the United States, the National School Lunch Program (NSLP) offers a unique opportunity to intervene at the population level on fruit and vegetable consumption as the program is in nearly 95% of primary and secondary schools [4],[5]. Efforts have increased over the past decade to capitalize on this opportunity and improve fruit and vegetable consumption among NSLP participants. In spite of a recent national school lunch policy requiring students to select a fruit or vegetable [6], elementary students are still throwing away 30–60% of the fruits and vegetables they select during lunch [7],[8]. Further, a multitude of school-based interventions targeting fruit and vegetable intake, ranging from nutrition education to systems and environmental change, have led to limited improvements among elementary school students, particularly with vegetables [9]. Additional behavior change strategies are needed to move the dial on dietary habits of elementary school-aged children.

One potential behavior change strategy that has not been used in a school cafeteria setting, to the authors' knowledge, is adult role modeling. Research in home settings suggests that modeling and encouragement from adults may influence child dietary intake [10]–[13]. Studies show that children are more likely to consume healthy foods when their parents and other adults consume healthy foods [10]–[13]. Similarly, fruit and vegetable consumption appears to be higher among children whose parents encourage fruit and vegetable consumption compared to children whose parents do not encourage such behavior [11],[12]. Little is known, however, about how adult role modeling can be leveraged to promote student fruit and vegetable consumption in school cafeteria settings. Drawing on Social Learning Theory [14], we hypothesize that adult consumption and promotion of the fruits and vegetables served in the NSLP will encourage students to consume more fruits and vegetables at lunch. Role models are routinely used in schools to address academic issues and behavioral problems [15],[16]. This study serves as an initial step toward evaluating role modeling in schools as a paradigm for health behavior change. The purpose of this pilot study is to evaluate the acceptability and initial outcomes of an adult role modeling intervention on lunchtime fruit and vegetable consumption among elementary school students participating in the NSLP.

## 2. Materials and methods

### 2.1. Intervention description

This one-year, repeated cross-sectional pilot study was conducted with kindergarten through fifth grade students in a low-income, ethnically diverse elementary school in rural Oregon. This study was deemed non-human subjects research by the Institutional Review Board at Oregon Tech as no identifiable information was collected. Researchers obtained approval from administrators at the elementary school before conducting the study.

Approximately 400 students were enrolled in the elementary school, with roughly 90% of children qualifying for free or reduced-price meals through the NSLP and 57% identifying as non-White. Two male college students, referred to as Cafeteria Role Models (CRMs) in this study, implemented the intervention over the course of school year 2016/2017. CRMs were selected based on their interest in the intervention and not based on their gender. CRMs interacted with students in the cafeteria 2–3 times per week for 20 weeks: 10 weeks in fall semester and 10 in spring semester. During each lunch period, CRMs ate lunch with kindergarten through fifth grade students, verbally promoted fruits and vegetables, and provided social reinforcement of fruit and vegetable consumption. Students rotated through the cafeteria in approximately 20-minute intervals over the course of the 1.5 hour lunch period, enabling CRMs to reach each lunch table and interact with most students each day.

The intervention is grounded in Social Learning Theory (SLT). This theory posits that the social environment influences both cognition and behavior; individuals observe a new modeled behavior, process the behavior, replicate the behavior, and are motivated to continue replicating the new behavior due to continued social reinforcement [14]. Students observe the adults eating fruits and vegetables and in turn replicate this behavior. They are motivated to continue eating the fruits and vegetables through continued social support from the adults and their peers.

**Table 1.** Role modeling intervention activities, fall & spring semesters.

Daily Role Modeling Activities	Daily Incentives	Short-Term Incentives	One-Time Incentives
			
(Fall & Spring Semesters)	(Fall & Spring Semesters)	(Over 2-week period in Spring Semester)	(End of Spring Semester)
Consume school lunch in cafeteria Consume all selected fruits and vegetables Verbal encouragement	Verbal praise High fives Fist bumps Hand stamps	Entry into raffle for classroom smoothie party Entry into raffle for student-designed, fruit and vegetable “Superhero” t-shirt	Fruit/vegetable smoothie party for one classroom T-shirts for 50 students

Role modeling activities in the intervention are based on two SLT components: observation and social reinforcement. Observation activities include: (1) CRMs eating school lunch; (2) CRMs selecting and eating all of their school-lunch fruits and vegetables; and (3) explanations by CRMs to students about what motivates the CRMs to eat fruits and vegetables. Social reinforcement activities included the following for students who tried at least one bite of their fruits and vegetables: (1) verbal praise, high fives, fist bumps, or hand stamps from CRMs; (2) raffle tickets administered by CRMs; and (3) raffle prizes given to students by CRMs. Observation activities were chosen based on encouragement and role modeling literature [10]–[13]. Social reinforcement activity 1 was chosen based on prior CRM experience working in school and after-school settings, and activities 2–3 were chosen based on input from the school's leadership students. Students in the leadership course assisted the CRMs in selecting the raffle prizes. Based on input from leadership students on what their peers would enjoy the most, the CRMs selected a classroom smoothie party and a raffle for a fruit and vegetable “superhero” t-shirt that a student in the leadership course designed. A complete description of the intervention activities are shown in Table 1. Each day that CRMs were in the cafeteria with students, they performed every role modeling activity and selected one daily incentive to give the students. Daily incentives were replaced with short-term incentives during a 2-week period in the spring semester. During this 2-week period, students received a raffle ticket for either a smoothie party or a t-shirt, depending on the day. Raffle tickets for the smoothie party were collected by classroom teachers. The classroom with the most raffle tickets at the end of the 2-week period were awarded a smoothie party. Further, individual students with the most t-shirt raffle tickets at the end of the 2-week period were awarded a t-shirt. The one-time incentives, a smoothie party and t-shirts, were given to students outside of the cafeteria setting.

## 3. Data collection and analysis

### 3.1. Process feedback

Cafeteria Role Models provided bi-weekly updates to the research team on their perceptions of intervention acceptability by students and staff. Perceptions were grounded in the CRMs' experiences gaining administrative approval for the intervention, delivering the intervention to students, and in soliciting intervention input from the leadership class students and their teacher.

### 3.2. Plate waste

To assess the impact of role modeling on fruit and vegetable consumption, the research team gathered information on plate waste. Plate waste assessments were conducted over eight school days at baseline (Spring 2016) and four days at follow-up (Spring 2017) to assess change in student fruit and vegetable consumption over the course of the pilot intervention. Staffing resource constraints prevented the research team from conducting an equal amount of days at follow-up. The pilot study, however, is moderately powered (0.55–0.72; p = 0.05) to detect approximately a 10% difference in waste, based on a test of two proportions. Plate waste was assessed over multiple days to account for variability in intake across different fruit and vegetable offerings and different days of the school week. Data was collected at the same time each year to account for seasonal variability in intake.

The assessments were conducted using a digital photograph method: a practical and cost-effective plate waste assessment method that has been validated amongst adult populations [17]. Digital photography is commonly used to assess plate waste in a school setting [18]. This method was chosen as an off-site assessment technique was necessary to mitigate cafeteria disruption. Researchers assessed the lunch tray of every 5^th^–7^th^ student in line to ensure a random sample of students and to minimize line slowdowns. Researchers assessed trays of kindergarten through 5^th^ grade students. Students were required to select one or more of the 1–2 fruit or 1–2 vegetable offerings. These offerings were pre-portioned in approximately ½ cup servings by cafeteria staff. Table 2 shows the types of fruits and vegetables offered at baseline and follow-up. At baseline and follow-up, three fruit and three vegetable offerings were the same. A greater variety was offered at baseline as more days of data were collected (8 days versus 4 days). Once students selected their food and exited the cafeteria line, two researchers put numbered stickers on student trays and took “before” photos of the trays. Two researchers took “after” photographs on cell phones and digital cameras while students waited to return their trays at the end of the lunch period. Additional researchers monitored the cafeteria to prevent students from trading trays or throwing away their food before an “after” picture was taken. Researchers uploaded the photos to a computer off-site and individually assessed, to the nearest 25%, the proportion of fruits, vegetables, and entrée that remained on each tray. Researchers received training and a written protocol on how to take photographs and assess quarter waste. Mann-Whitney statistical tests were used to assess differences in fruit and vegetable intake between baseline and follow-up. This nonparametric model was used to accommodate the non-normal distribution of residuals and the large proportion of 0 values. Statistical tests were conducted using STATA 15 software.

**Table 2.** Fruits and vegetable offerings, baseline and follow-up.

Spring 2016		Spring 2017	
Fruits	Vegetables	Fruits	Vegetables
apples	green salad	apples	green salad
assorted canned fruit	carrots	assorted canned fruit	carrots
canned pineapple	broccoli	canned pineapple	broccoli
canned cranberries	peas	canned pears	cucumbers
raisins	corn	bananas	celery
kiwi	cauliflower		
blueberries	green beans		

## 4. Results

### 4.1. Process feedback

Staff reported high levels of intervention acceptance. The principal commented that the role modeling aspect of the intervention was valuable for students because the school had a goal of promoting college attendance. The principal noted an additional value in this role modeling intervention, beyond improving dietary intake or reducing waste, as elementary school students had the opportunity to develop relationships with the college student CRMs delivering the intervention. The CRMs were male, an under-represented mentor group at the school, which proved to be an asset for the school and an added benefit of the intervention. Further, the leadership teacher reported acceptance of the intervention as it provided a project for which her students to engage. CRMs reported that students enjoyed receiving both weekly incentives and raffle entries. Students also developed relationships with the CRMs and seemed to enjoy conversing with them. The established relationship between college-aged researchers and students appeared to be pivotal in the acceptance of both the intervention aimed at improving dietary behavior and the support received from teachers and administration within the school.

### 4.2. Pilot outcomes

Data from 566 trays at baseline and 231 trays at follow-up were collected (approximately 70 trays per day). Table 3 presents the distribution of plate waste for fruits, vegetables, and the total plate (entrée, fruits, and vegetables) at baseline and follow-up. Generally, the results suggest no increase in fruit and vegetable waste and a decrease of complete fruit waste. The extremes, complete waste and zero waste were the areas in which change was observed. At baseline, 33.9% of students wasted all (i.e. consumed none) of their selected fruits, compared to only 17.9% at follow-up. This 16.0% decrease in the proportion of students consuming no fruit was statistically significant (p < 0.001). Further, at baseline, 38.7% of students consumed all (i.e. wasted none) of their selected fruits, while at follow-up 49.8% of students consumed all of their fruit demonstrating an 11.1% difference (p < 0.01).

**Table 3.** Percentage of plate waste of fruits, vegetables, and total plate at baseline and follow-up (n = 797).

% of Waste	Fruit^a^		Vegetable^a^		Total^b^	
	Baseline	Follow-up	Baseline	Follow-up	Baseline	Follow-up
	N = 437	N = 207	N = 566	N = 231	N = 566	N= 231
0% (no waste)	38.7%	49.8%**	18.6%	27.3%**	15.7%	17.8%
25%	8.7%	5.8%	6.5%	3.9%	25.6%	32.9%
50%	9.8%	15.0%	5.5%	10.8%	26.7%	23.4%*
75%	8.9%	11.6%	7.4%	7.8%**	27.2%	24.2%
100% (complete waste)	33.9%	17.9%***	22.1%	26.4%	3.7%	1.7%

Note: ^a^All plates included a fruit at Baseline and Follow-up. Approximately 60% of plates included a vegetable at Baseline compared to 76% at Follow-up. The difference was statistically significant at the p < 0.01 level. ^b^Fruit, vegetable, and entrée; *indicates a statistically significant differences between Baseline and Follow-up at the p < 0.05 level; **indicates a statistically significant differences between Baseline and Follow-up at the p < 0.01 level; ***indicates a statistically significant differences between Baseline and Follow-up at the p < 0.001 level. Differences at Baseline and Follow-up were assessed using a Mann-Whitney Test (Two-Sample Wilcoxon Rank-Sum).

Additionally, there was a significant increase in the proportion of students consuming all of their selected vegetables. Approximately 18.6% of students in the study consumed all of their selected vegetables at baseline compared to 27.3% of students at follow-up, representing approximately an 8.7% difference (p < 0.01). At follow-up, a slight increase of 0.4% was found in the 75% waste category (p < 0.01). Further, from baseline to follow-up there was a 16.0% increase (p < 0.01) in the proportion of students selecting a vegetable. Approximately 60% of plates included a vegetable at baseline compared to 76% at follow-up. Total plate waste at the 50% level decreased by 3.0% (p < 0.05). No other significant changes were observed.

## 5. Discussion

This pilot offers promising support for the acceptability and potential efficacy of role modeling interventions to promote fruit and vegetable consumption. Informal conversations with the school principal point to the alignment of the intervention with school goals as a contributor to high levels of staff acceptance of the intervention. The intervention dovetailed with the school's goal of promoting college attendance. This supports the findings by Brownson et al. [19] that interventions aligning with the goals of the organization in which they are implemented are more likely to be successful than those without alignment. This is of particular importance for public health initiatives in non-health settings. Additionally, the low-cost incentives that were used in the intervention were well-received by students, demonstrating that incentives/rewards may not need to be high-cost in order to be effective.

Pilot results point to the potential viability of adult role modeling to improve fruit and vegetable consumption and reduce waste among elementary school-aged children. Data shows that, in general, the majority of students in the sample waste either all or none of their fruits and vegetables. At follow-up, pilot data shows significant increases in the proportion of students consuming all of their selected fruits and vegetables and a significant decrease in the proportion of students not consuming any of their selected fruit. Further, there was a significant increase in the proportion of students selecting a vegetable. Increases in fruit and vegetable consumption did not appear to influence entrée intake. These findings offer support for the theoretical assumption that adult role modeling behavior may influence student dietary behavior. Role modeling behavior has been used in a variety of different educational contexts [15],[16], and findings from this study suggest role modeling may be a viable method to reduce waste and increase healthy eating among students.

Furthermore, role modeling serves as a low-cost method to address a spectrum of behaviors within the school context. A primary advantage to using role models in a school setting is the flexibility of direction. The basis for successful relationship-development between a role model and a child is trust [20]. Once this foundation is built, many behaviors can become modeled. In the context of this study, the CRMs could have switched the focus from fruit and vegetable consumption to participation in science, increasing physical activity, or processing and dealing with emotional or behavioral issues. The advantage of utilizing role models in schools is that CRMs can support efforts to address multiple behaviors, in multiple roles, and in multiple school settings ranging from the cafeteria to the gym or the classroom. Because role modeling can be so versatile safeguards must also be in place to ensure the behavior being modeled is appropriate and the consequences are positive. This requires training and mentorship of CRMs and oversight and input from the school or organization. This study suggests that building a partnership between the research team and school administration greatly benefits the implementation of role model-based interventions.

This initial study serves as an important step in the process of understanding the most effective strategies to promote health in schools. Although the study is a pilot, there is now some empirical support for the theoretical framework. Moving forward, more robust study design components are needed to determine the extent to which role modeling can serve as an effective tool for encouraging fruit and vegetable consumption. First, a future study should include a comparison group. The lack of a comparison group makes it difficult to assess whether changes in consumption can be attributed to the intervention or to another source. The research team, however, did not observe other school-level changes that appeared to have influenced fruit and vegetable consumption. Further, participant demographics were not collected due to feasibility considerations. Future studies should collect this information in order to adjust for gender or age: known moderators of fruit and vegetable consumption [12]. Further, as power calculations are difficult to compute for non-parametric statistical tests, we used standard methods for power with a dichotomized outcome, as is traditionally done [21]. This calculation may under-estimate power [22]. Though only moderately powered, we were still able to detect significant differences in intake, indicating our effect sizes are quite large. Future studies should be adequately powered to detect smaller differences in intake.

Finally, as this was a pilot with no funding for evaluation activities, resource constraints limited our ability to collect systematic process data, assess role modeling mechanisms, or quantify the amount of fruits or vegetables, in cups or grams, wasted. In scaling up, future studies should collect systematic data on process measures, such as fidelity, dose, and acceptability, to describe implementation effectiveness [23]. Further an assessment of specific role modeling behaviors should also be undertaken to understand which specific components of role modeling are associated with student fruit and vegetable consumption. Finally, future studies should employ a more rigorous plate waste assessment method that includes between-researcher comparisons of plate waste codes to mitigate coding discrepancies, the matching of menus at baseline and follow-up based on the type and preparation of fruits and vegetables served, and reports on amounts of fruits and vegetables selected and consumed. Additional studies are needed to test the acceptability and efficacy of this pilot intervention at a larger scale.

## 6. Conclusions

This pilot study is the first to explore if adult role modeling influences student fruit and vegetable consumption in an elementary school cafeteria setting. While adults in the home can positively influence child dietary habits [10], findings from this study suggest that adults in a school setting may also play a role in positively influencing student dietary behavior through modeling and incentivizing fruit and vegetable consumption. Role modeling may serve as an approach to promote healthy behaviors. Schools can take advantage of positive social interaction in building a healthier generation.
